# The Relationship between Live Sports Learning and Exercise Behavior in College Students: A Serial Mediation Model

**DOI:** 10.3390/bs14040266

**Published:** 2024-03-23

**Authors:** Tiantian Guo, Liping Liu, Yuqing Yang, Yao Shang, Shan-Ping Chen

**Affiliations:** Center for Physical Education, Xi’an Jiaotong University, Xi’an 710049, China; tiantianguo@stu.xjtu.edu.cn (T.G.); shangyao@xjtu.edu.cn (Y.S.); chshp@xjtu.edu.cn (S.-P.C.)

**Keywords:** live sports streaming, online learning, exercise behavior, exercise motivation, exercise commitment, college students

## Abstract

Physical exercise is crucial to the development of students’ physical and mental health. This study explored the relationship between live sports learning and college students’ exercise behaviors, and the mediating roles of exercise motivation and exercise commitment, aiming to provide theoretical bases for the future that explain the mechanism of live sports learning in exercise behaviors, as well as practical guidance for the promotion of positive physical exercise behaviors in college students. In total, 1189 college students from China volunteered to complete questionnaires. The results showed that live sports learning positively predicted college students’ exercise behavior and that live sports learning was able to affect exercise behavior through the mediating roles of exercise motivation and exercise commitment, with specific mediating paths including the two independent mediating paths and a serial mediating path of exercise motivation and exercise commitment. This study confirmed, for the first time, on live sports learning in the process of promoting exercise behavior. It is suggested that educators instruct college students to regulate their participation in live sports learning and to cultivate healthy exercise motivation and exercise commitment, which is an effective way to facilitate college students’ practice of physical activity.

## 1. Introduction

Insufficient physical activity leading to a decline in physical fitness has emerged as a severe global public health issue [1]. In response to the existing health issues, countries and regions worldwide have repeatedly emphasized the importance of fostering good exercise habits and promoting the overall development of students’ physical and mental well-being [2]. Extensive research demonstrates the positive effects of physical exercise on both the body and mind [3,4,5]. However, studies reveal that the current state of physical exercise and physical fitness among college students is far from satisfactory [6]. College students represent the future driving force of social development; establishing and maintaining exercise behaviors during this period significantly influence the development of self-initiated exercise awareness and lifelong physical education perspectives in adulthood [7]. Therefore, promoting active participation in physical exercise among college students and enhancing their physical and mental well-being remain key focal points of scholarly attention.

In recent times, online learning models have been iterated and upgraded with the help of information technology [8]. Live sports learning refers to the online learning behavior of students who invest time and effort in participating in live sports streaming to acquire sports skills and knowledge related to physical activities. Types of live sports learning encompass online fitness live streaming [9], sports event live streaming [10], physical education live streaming [11], etc. Learning sports-related knowledge through live sports streaming among college students has become increasingly popular. Importantly, it is more effective than traditional learning methods in enhancing users’ sports skills and fostering interest in achieving learning outcomes [8]. Studies have shown that videos about the technical abilities used in soccer [12], football [13], and volleyball [14] are receiving much attention from participants. Additionally, live sports streaming provides access to professional sports competitions [15], team analysis [16], and relevant knowledge about sports injury treatment [17]. It is evident that participating in live sports learning has become a popular way for college students to engage in online education, as well as a form of leisure and entertainment, attracting scholarly attention due to its substantial number of participants. Now, we have a question. Is college students’ engagement in live sports learning effectively promoting exercise behavior? This aspect deserves to be explored in depth.

Currently, considerable progress has been made in investigating the factors influencing college students’ exercise behavior. However, it is evident that the academic community still lacks in-depth research on the influence of live sports learning and exercise behavior, as well as a comprehensive examination of the mediating effect of the “exercise motivation and exercise commitment” chain. In light of this, our study aims to address this gap by focusing on the scenario of college students’ participation in live sports learning. It aims to construct a theoretical path model that explores the effect of live sports learning on exercise behavior and investigates the relationship between these two variables, while also examining the mediating effects of exercise motivation and exercise commitment. This research will not only provide valuable theoretical support for further exploration of live sports learning and physical activity education but will also have practical implications for enhancing college students’ enthusiasm and effectiveness in engaging in physical exercise.

## 2. Literature Review and Research Hypothesis

### 2.1. Relationship between Live Sports Learning and Exercise Behavior

Existing studies have explored both the positive and negative impacts of live sports learning, with current studies largely focusing on its negative effects. When students are unable to use the internet responsibly, it can lead to increased feelings of loneliness [18] and a decline in academic performance [19]. However, it is worth noting that scholars have also highlighted the positive impact of live sports learning. Research revealed that participating in online fitness live streaming can enhance intentions for physical exercise [9], and the fan effect among users of sports live platforms can stimulate sports consumption behavior [20]. Therefore, the model in our study aims to affirm the positive nature of live sports learning.

The self-determination theory is one of the most successful theoretical models for understanding and predicting exercise behavior [21,22,23]. If an individual perceives exercise as voluntary, consistent with their values, and capable of fulfilling their intrinsic needs, they are more likely to sustain regular exercise. Online live streaming creates a favorable digital learning space, catering to the diverse learning needs of students [24]. The self-determination theory can also be applied to explain the relationship between live sports learning and exercise behavior. Empirical research has found that meeting psychological needs is a key factor in promoting physical exercise [22,23]. When students feel that live sports learning can fulfill their diverse needs for sports education, they are more likely to maintain a positive attitude toward exercise [25]. Renowned researchers in exercise psychology have identified the personal factors influencing exercise behavior. They have constructed the Mechanism Model of Exercise Persistence, which has confirmed the positive role of personal investment in exercise behavior [26]. Their research findings also indicate that more personal investment is associated with a higher likelihood of engaging in physical exercise [27]. Live sports learning involves both a personal investment of time and energy, and may also include a personal financial investment of monetary expenditures. College students are enthusiastic about participating in live sports learning and personally invest time and energy in online learning to gain knowledge about sports skills and physical activity [12,13,14,15,16,17]. Therefore, participation in live sports learning effectively enhances their loyalty to sports activities [28]. Based on this, the research hypothesis is proposed:

**Hypothesis (H1).**  *Live sports learning positively predicts exercise behavior in college students.*

### 2.2. Exercise Motivation as a Mediator

Psychological research categorizes motivation into intrinsic motivation and extrinsic motivation [29,30]. Our study focuses on intrinsic motivation for exercise, which refers to the psychological drive of individuals to engage in exercise based on their own interests or internal needs. As a core element of the psychological decision-making process, exercise motivation is closely linked to live sports streaming and exercise behavior. Firstly, several research findings in the field of education suggest that online learning enhances students’ motivation and interest in learning [31,32], and live sports learning attracts college students who are passionate about physical exercise, contributing to the enhancement of their exercise motivation. Furthermore, psychologist Ryan’s [33] research proposes that individuals with stronger intrinsic motivation for physical exercise are more likely to persist in exercising. Studies consistently indicate that exercise motivation serves as a predictor of exercise behavior [34,35,36]. Some scholars have identified exercise motivation as a mediating variable, confirming its ability to improve exercise behavior [27,37]. Therefore, exercise motivation has a mediating role, and it can be inferred that live sports learning can stimulate college students’ exercise motivation and promote their exercise behavior. Based on this, the research hypothesis is proposed.

**Hypothesis (H2).**  *Exercise motivation has a mediating role between live sports learning and exercise behavior in college students.*

### 2.3. Exercise Commitment as a Mediator

In the field of sports research, exercise commitment refers to the psychological state of individuals who have a desire and determination to continue engaging in physical exercise [38], manifested as their behavioral intention toward exercise [39]. Firstly, there is a close relationship between live sports learning and exercise commitment. A study indicates that the content of online fitness broadcasts is a crucial factor influencing the exercise commitment of the audience [9], and online viewing of sports videos has a positive influence on exercise commitment [40]. Therefore, the content of sports broadcasts can engage college students actively, fostering the generation of exercise commitment. Secondly, exercise commitment is a determinant factor for exercise behavior. According to the exercise commitment theory [41], commitment is an individual’s intention or plan for a target behavior, guiding individuals in choosing how to execute future actions. College students invest time and effort in participating in live sports learning and acquiring knowledge related to sports health and skills, which can guide individual exercise intentions toward a healthy and positive direction, and encourage the formation of positive exercise behaviors [13,17]. Research has also clarified that exercise commitment, as a mediating variable, can effectively enhance the engagement of individuals in physical activities [42]. Therefore, exercise commitment plays a mediating role, and it can be inferred that live sports learning can positively influence college students’ exercise commitment, thereby promoting exercise behavior. Based on this, the following research hypothesis is proposed.

**Hypothesis (H3).**  *Exercise commitment has a mediating role between live sports learning and exercise behavior in college students.*

### 2.4. Exercise Motivation and Exercise Commitment as Serial Mediators

Research exploring the psychological mechanisms of exercise behavior has identified a significant connection between exercise motivation and exercise commitment [43], and exercise motivation is a stimulating source of exercise commitment [39,44]. According to cognitive psychology, individual cognition influences target behavior by acting on mental decisions, and this viewpoint has been specifically interpreted in the Mechanism Model of Exercise Persistence [27]. In other words, individual factors, as cognitive variables, influence exercise persistence by acting on exercise motivation and exercise commitment. Recent studies have found that college students’ exercise identity, as an important individual factor, can enhance the psychological decision-making of exercise commitment by stimulating exercise motivation, which in turn positively affects exercise behavior [45]. In the various studies on the mechanism of factors’ influence on exercise behavior, exercise motivation and exercise commitment play independent chain-mediated roles [45,46]. Thus, when considering the effect of live sports learning on exercise behavior, it is essential to undergo a cognitive evaluation of exercise motivation, followed by a feasibility assessment of exercise commitment, before making an informed decision to engage in physical exercise. Based on these findings, the following research hypothesis is proposed:

**Hypothesis (H4).**  *Exercise motivation and exercise commitment have a serial mediating role between live sports learning and exercise behavior in college students.*

**Figure 1:**
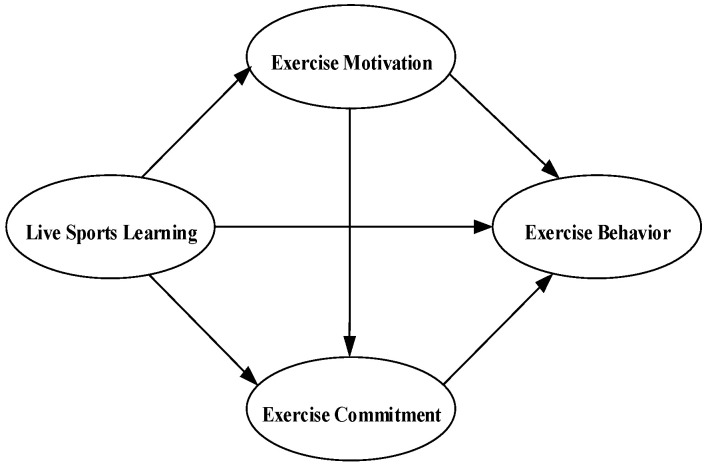
Hypothesis model.

Based on the hypotheses (H1, H2, H3, and H4), we propose a serial mediation model that integrates live sports learning, exercise motivation, exercise commitment, and exercise behavior (shown in Figure 1).

## 3. Materials and Methods

### 3.1. Participants and Procedures

This study was approved by the Biomedical Ethics Committee of Xi’an Jiaotong University Health Science Center based on the Declaration of Helsinki (No. 2022-1685). Participants signed an informed consent form. Participants were recruited from comprehensive universities in China. The sample data were collected through the “Questionnaire Star” online platform. The questionnaire guide provided detailed information about the research purpose and participation conditions (voluntary and confidential). The formal survey was conducted from 3 December 2022 to 9 December 2022, using a quota sampling method based on gender and grade-level categories. In total, 1231 questionnaires were collected, and 1189 valid questionnaires were retained that were in accordance with the screening principles of the regularity of filling in the answers and an answering time of less than 150 s, with an effective recovery rate of 96.59%. Among the participants, there were 680 males and 509 females, with 334 freshmen, 298 sophomores, 280 juniors, and 277 seniors.

### 3.2. Instruments

#### 3.2.1. Live Sports Learning

In accordance with the description of sports consumption from Dai [47], after a comprehensive review of the literature and practical research considerations, we compiled our questionnaire to investigate college students’ participation in live sports learning. This was in accordance with the backdrop of live sports learning and the specific objectives of our research. The 5 types of live sports learning correspond to 3 dimensions of participation frequency, participation duration, and consumption amount, with a total of 15 questions. Types of live sports learning encompass online fitness live streaming, sports event live streaming, physical education live streaming, e-sports live streaming, and sports e-commerce live streaming [48]. Questionnaire items are shown in Appendix A. The score for participation frequency is the sum of scores from participating in the five types of live sports streaming. The score for participation duration is the sum of scores from participating in the five types of live sports streaming. The score for the consumption amount is obtained by summing up the expenditure in the five types of live sports streaming and is normalized using the following calculation formula: ln X ≈ ln(1 + X) [49].

Before the formal survey, a content validity assessment of the measurement items was conducted using the expert assessment method [50]. Eight authoritative experts were invited to evaluate the content validity of the questionnaire items. The calculation results revealed that the item–content validity index (I-CVI) ranged from 0.875 to 1.00, all of which exceeded the minimum standard of 0.70. The scale–content validity index (S-CVI) was 0.937, surpassing the minimum standard of 0.80. These findings indicate the favorable content validity of the questionnaire [50]. In this study, Cronbach’s α was 0.814, showing the great interior consistency of the scale.

#### 3.2.2. Exercise Motivation

The Motivation for Physical Activities Measure (MPAM-R), initially developed by Ryan [33] and subsequently revised by Chen [51], was employed to assess the intrinsic motivation for exercise among college students, with a total of 15 questions including 5 dimensions: health motivation, appearance motivation, fun motivation, social motivation, and ability motivation. Scale items are shown in Appendix A. The five-point Likert-type scale was used, each subscale consisted of 3 questions, the subscale score was the average of the 3 questions, and the total exercise motivation score was the average of the 5 subscale scores. Cronbach’s α of the total scale was 0.737, and Cronbach’s α of each sub-scale is 0.725~0.899 [51]. In this study, Cronbach’s α of the total scale was 0.916, and Cronbach’s α of each sub-scale was 0.868~0.949.

#### 3.2.3. Exercise Commitment

The Exercise Commitment Scale, based on the Mechanism Model of Exercise Persistence developed by Chen [27], was employed to evaluate participants’ psychological dispositions, reflecting their willingness and determination to partake in physical exercise. There were four questions. Scale items are shown in Appendix A. The five-point Likert-type scale was used. The total exercise commitment score was averaged across the four questions. The internal consistency coefficient of exercise commitment is 0.958 [27]. Cronbach’s α in this study was 0.947.

#### 3.2.4. Exercise Behavior

The Physical Activity Rating Scale (PARS-3) was introduced and revised by Liang [52] from Wuhan Sports University in China. This scale has seen broad acceptance among researchers as a tool for evaluating the physical activity levels of students. There are 3 questions including exercise time, weekly frequency, and intensity of physical activity. Scale items are shown in Appendix A. The five-point Likert-type scale was used. The total physical exercise level score was calculated using the following formula: (exercise time score − 1)  ×  exercise frequency score  ×  exercise intensity score. This scale has been widely used in China; in this study, Cronbach’s α was 0.728, showing the great interior consistency of the scale.

### 3.3. Statistical Analyses

All statistical analyses were conducted using the IBM SPSS Statics 26.0 program and IBM SPSS Amos 25.0 software. Firstly, descriptive statistics, an internal consistency reliability test, and Pearson’s product–moment correlation were conducted in SPSS. Secondly, Anderson and Gerbing’s [53] two-stage method was used to analyze the data. Confirmatory factor analysis was used to examine the measurement tool’s psychometric properties, and structural equation modeling procedures, with the maximum likelihood approach, were used to examine the structural relationships between the proposed variables through Amos, while the bootstrap method was used to test the mediation effect.

Specifically, a set of fitting indices was considered for the model fit: a goodness of fit index (GFI) >  0.90, comparative fit index (CFI) >  0.90, incremental fit index (IFI) >  0.90, normed fit index (NFI) >  0.90, Tucker–Lewis index (TLI) >  0.90, and root mean square error of approximation (RMSEA)  <  0.08 [54]. The bootstrap method was set to draw a sample of 5000 at 95% confidence intervals (CIs) [55].

## 4. Results

### 4.1. Correlation Analysis and Reliability Validity Analysis

As shown in Table 1, the correlation analysis reveals significant positive correlations among live sports learning, exercise motivation, exercise commitment, and exercise behavior. The correlation coefficients ranged from 0.151 to 0.590 at the *p* < 0.01 significance level. These significant correlations provide the foundation for the further testing of effects between the research variables.

This study utilized confirmation factor analysis to verify the measurement model. The results showed that the model fitting indexes were χ^2^ = 357.951 and DF = 84, χ^2^/DF = 4.261, meeting the criterion of being less than 5. GFI = 0.961, CFI = 0.978, IFI = 0.978, NFI = 0.972, and TLI = 0.973, all exceeding the criterion of being greater than 0.9. RMSEA = 0.052, below the criterion of being less than 0.08 [54]. These results indicate a good fit for this model. In the measurement model, reliability and validity analyses are shown in Table 1, and for the assessment of internal consistency within the scale, Cronbach’s α coefficient meets the standard of 0.7 or above [56]. Composite reliability (CR) ranges from 0.756 to 0.946, meeting the criterion of being greater than 0.7 [53]. The average variance extracted (AVE) falls between 0.514 and 0.816, adhering to the standard of being greater than 0.5 [53]. Lastly, discriminant validity is confirmed when the square root of the AVE ($\sqrt{AVE}$) is greater than the correlation coefficients with other latent variables [56]. The correlation coefficients between variables range from 0.151 to 0.590, and the $\sqrt{AVE}$ for each variable is greater than its correlation coefficients with other variables. Overall, the results demonstrate that the instruments used in this study have good reliability and validity.

### 4.2. Hypothesis Testing

**Figure 2:**
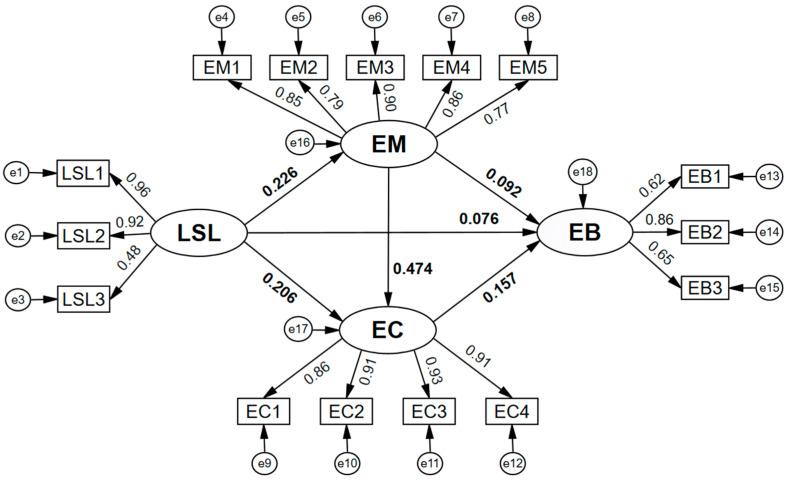
Schematic diagram of path analysis of LSL, EM, EC, and EB. LSL, live sports learning; EM, exercise motivation; EC, exercise commitment; EB, exercise behavior. LSL1, LSL2, and LSL3 represent participation duration, participation frequency, and consumption amount, respectively; EM1, EM2, EM3, EM4, and EM5 represent health motivation, appearance motivation, fun motivation, social motivation, and ability motivation, respectively; EC1, EC2, EC3, and EC4 are the 4 questionnaire items for exercise commitment; EB1, EB2, and EB3 represent exercise time, exercise frequency, and exercise intensity, respectively.

The results show that the structural model in the study has a good fit: χ^2^ = 357.951, DF = 84, χ^2^/DF = 4.261, GFI = 0.961, CFI = 0.978, IFI = 0.978, NFI = 0.972, TLI = 0.973, and RMSEA = 0.052 [54]. A specific path analysis schematic diagram is shown in Figure 2. As shown in Table 2, the regression coefficient of live sports learning on exercise behavior was significant (r = 0.076, *p* < 0.020, supporting hypothesis H1), indicating that participation in live sports learning had a significant positive effect on exercise behavior. In addition, the regression coefficients of live sports learning on exercise motivation (r = 0.226, *p* < 0.001) and exercise commitment (r = 0.206, *p* < 0.001) were significant. The regression coefficients of exercise motivation on exercise behavior (r = 0.092, *p* < 0.030) and exercise commitment (r = 0.474, *p* < 0.001) were significant. The regression coefficient of exercise commitment on exercise behavior (r = 0.157, *p* < 0.001) was significant.

The bias-corrected percentile bootstrap method (repeated sampling 5000 times) was used to test the mediating effect of exercise motivation and exercise commitment in the relationship between live sports learning and exercise behavior, and the test results are shown in Table 3. The mediated path of live sports learning affects exercise behavior via exercise motivation, the mediated path of live sports learning affects exercise behavior via exercise commitment, and the chain-mediated path of live sports learning affects exercise behavior via exercise motivation and exercise commitment. The above three paths are within the bias-corrected and percentile 95% intervals (the intervals do not include 0) [55], indicating that the mediating effects are significant. Support for H2, H3, and H4 is based on the above analysis.

## 5. Discussion

The current study proposed and tested a serial mediation model to examine the relationships among live sports learning, exercise motivation, exercise commitment, and exercise behavior in a sample of Chinese college students. The findings of the present model are consistent with our hypotheses. Live sports learning positively predicts exercise behavior, exercise motivation has a mediating role between live sports learning and exercise behavior, exercise commitment has a mediating role between live sports learning and exercise behavior, and we also found that exercise motivation and exercise commitment have a serial mediating role between live sports learning and exercise behavior in college students.

### 5.1. Live Sports Learning Helps Promote Exercise Behavior

Our research findings indicate that live sports learning contributes to the promotion of exercise behavior. In alignment with numerous prior investigations, live sports learning has been consistently associated with fostering engagement in sports activities [28,57], boosting fitness intentions [9], increasing involvement in sports consumption [20], and acquiring sports-related knowledge [15]. From a psychological perspective, the greater the individual’s investment, the higher the likelihood of maintaining exercise [27]. College students, equipped with the ability for online learning, invest time and energy into sports live learning, which serves as a novel avenue for them to acquire sports skills and knowledge. The evidence presented above supports the positive predictive relationship between live sports learning and exercise behavior. Through a cross-sectional study, our research also demonstrates that dedicating time and effort to live sports learning has a direct impact on exercise behavior, providing applicable insights for promoting physical exercise among college students engaged in live sports learning.

### 5.2. A Serial Mediating Role of Exercise Motivation and Exercise Commitment

Firstly, we discuss the mediating role of exercise motivation. Exercise psychology reveals that exercise motivation is a precursor factor that promotes college students’ exercise behavior [30,34]. Several studies have confirmed that exercise motivation is an important psychologically mediated variable in individual cognitive and behavioral influence mechanisms [27,30,34]. As described by the theory of cognitive decision, exercise motivation is regarded as a crucial component in the demand analysis phase of the cognitive decision-making process [27], where individuals evaluate their needs for live sports learning and engage in exercise activities accordingly. Additionally, in terms of sports software, research has found that sports applications can stimulate users’ needs for physical exercise, thereby positively influencing their exercise awareness and behavior [58]. Sports applications and live sports learning both operate on the internet and offer features such as fitness, social interaction, and information acquisition. In real-life situations, for example, students who are enthusiastic about live football matches may choose to invest their time and energy in streaming their favorite sports. This choice can trigger an evaluation of their exercise motivation for football or other sports, ultimately promoting the generation of exercise behavior. Our study conclusively confirmed the mediating role of exercise motivation in the process by which live sports learning affects exercise behavior, which precisely explains the reasoning described above.

Secondly, we discuss the mediating role of exercise commitment. Previous research has demonstrated that exercise commitment serves as a direct predicate variable to exercise behavior and functions as a psychological mediator variable in the cognitive–behavioral influence process [27,39]. Exercise commitment, defined as an individual’s behavioral intention [59], aligns with the theory of reasoned action, which suggests that behavioral intentions precede actual behavior and serve as reliable predictors of exercise behavior [60]. In accordance with previous research, our study contributes to the body of evidence affirming the explanatory and predictive significance of exercise commitment in individuals’ exercise behavior. Additionally, from the rise in live online fitness streaming during the COVID-19 pandemic, research on participation in online fitness live streaming has revealed that sports learning through live streaming can generate a strong sense of pleasure and positively affect individuals’ intention to engage in exercise [9]. In reality, exemplified by “Tiktok” sports live streaming, it has attracted a large number of users to participate in physical exercise, making participation in live sports learning a new form of fitness. Our study confirms the mediating effect of exercise commitment in the process of exercise behavior influenced by live sports learning. Therefore, hypothesis (H3) suggests that when college students concentrate and fully engage in live sports learning, it can enhance exercise commitment, subsequently fostering the occurrence of exercise behavior.

Lastly, the chain-mediated effect of exercise motivation and exercise commitment is observed. On the one hand, it is explained by the cognitive decision model of exercise persistence [27] mentioned above, where the simplified path in this model is “personal factors → exercise motivation → exercise commitment → exercise persistence”. This theory supports the findings of our study, where live sports learning serves as a perceived personal factor that affects exercise commitment through the cognitive decision-making process of exercise motivation, ultimately affecting exercise behavior. On the other hand, some scholars have applied the actor–critic theory from reinforcement learning to the study of exercise behavior prediction models [6]. They suggest that the decision-making process involves two computational processes: the evaluation of action value and the evaluation of action feasibility [61]. In the context of our study, exercise motivation corresponds to the actor, mainly involving the assessment of the value of engaging in exercise behavior by college students after perceiving live sports learning information. Exercise commitment corresponds to the critic, primarily determining the feasibility of the exercise behavior based on the evaluation results of exercise motivation. These explanations further emphasize the predictive role of exercise motivation and exercise commitment in exercise behavior. Therefore, it can be seen that college students’ exercise motivation and exercise commitment are essential pathways through which live sports learning affects exercise behavior.

### 5.3. Value and Limitations

For the first time, this study takes live sports learning as a predictor variable of college students’ exercise behavior, which complements the effect factors of the mechanism of exercise behavior, expands the effect path of exercise behavior, and provides a theoretical foundation for further in-depth understanding of the relationship between live sports learning and exercise behavior. At the same time, our study clarifies the value of live sports learning in the process of promoting exercise behavior and also confirms the mediating role of exercise motivation and exercise commitment in the influence of live sports learning on exercise behavior. Therefore, we suggest that educators in higher education can provide online learning information on physical education in the classroom and also on the Web. They should instruct students to regularly participate in live sports learning, guide students to acquire relevant knowledge about physical health and exercise skills, strengthen the cultivation of motivation and commitment to exercise, and enhance the positivity and normality of physical activity, which provides practical enlightenment for higher education.

As with any study, our study is not without its limitations. First, due to its cross-sectional design, it was difficult to conduct a causal analysis of the variables. Second, our study only considered the mediating role of exercise motivation and exercise commitment, and other variables of psychological decision-making should be considered comprehensively in subsequent studies to further improve the explanatory power of exercise behavior, for example, by incorporating social support variables from parents, teachers, and friends [62], or exploring variables such as exercise self-efficacy [63], exercise attitude [64], and subjective experience [39,42]. Finally, the participants of this study were college students, and in the context of National Fitness, we need to expand the research groups and study the current characteristics of students of different ages and other groups of people, further expanding the value of the study. It is worth noting that live sports learning is appealing to young persons, but this emerging way of promoting exercise is potentially challenging for older people. Therefore, exploring the benefits of older adults’ participation in live sports learning to help them enjoy a healthy life with good well-being is also a direction for future research.

## 6. Conclusions

The present study is unique in proposing live sports learning as a predictor variable of college students’ exercise behavior, and in proposing a chain mediation model to explain the relationship between live sports learning and exercise behavior. We also found that live sports learning positively predicted college students’ exercise behavior and that live sports learning was able to influence exercise behavior through the mediating roles of exercise motivation and exercise commitment, with specific mediating paths, including the two independent mediating paths and a serial mediating path of exercise motivation and exercise commitment. This study further expands the path of live sports learning in the process of promoting exercise behavior. It is suggested that educators incorporate online learning resources for physical education in higher education, instruct college students to regulate their participation in live sports learning, and cultivate healthy exercise motivation and exercise commitment, which is an effective way to facilitate college students’ practice of physical activity.

## Figures and Tables

**Table 1:** Correlation analysis and reliability validity analysis.

	$\mathrm{Correlation}\;\mathrm{Coefficients}\;\mathrm{and}\;\sqrt{AVE}$				Cronbach’s α	CR	AVE
	LSL	EM	EC	EB	>0.7	>0.7	>0.5
LSL	0.816 ^a^				0.814	0.848	0.666
EM	0.192 ***	0.836 ^a^			0.916	0.921	0.700
EC	0.196 ***	0.590 ***	0.903 ^a^		0.947	0.946	0.816
EB	0.156 ***	0.151 ***	0.165 ***	0.716 ^a^	0.728	0.756	0.514

*** *p* < 0.01. ^a^ indicates that data are the square root of the AVE of each variable. LSL, live sports learning; EM, exercise motivation; EC, exercise commitment; EB, exercise behavior.

**Table 2:** The regression weights between LSL, EM, EC, and EB.

Associations between Variables	Standardized Regression Weights (r)	S.E.	C.R.	*p*
LSL → EB	0.076	0.022	2.171	<0.030
LSL → EM	0.226	0.027	6.884	<0.001
LSL → EC	0.206	0.030	7.048	<0.001
EM → EB	0.092	0.030	2.328	<0.020
EM → EC	0.474	0.037	16.168	<0.001
EC → EB	0.157	0.025	3.859	<0.001

LSL, live sports learning; EM, exercise motivation; EC, exercise commitment; EB, exercise behavior.

**Table 3:** Bootstrap analysis of mediating effects test.

Path Relationship	Effect Size	Bootstrap SE	Bias-Corrected 95%CI		Percentile 95% CI	
			Lower	Upper	Lower	Upper
Total effect	0.146	0.036	0.076	0.218	0.075	0.217
Direct effect	0.076	0.036	0.006	0.150	0.003	0.147
Total indirect effect	0.070	0.013	0.047	0.098	0.046	0.096
LSL → EM → EB	0.021	0.009	0.004	0.040	0.003	0.039
LSL → EC → EB	0.032	0.010	0.016	0.054	0.016	0.053
LSL → EM → EC → EB	0.017	0.005	0.009	0.027	0.008	0.027

LSL, live sports learning; EM, exercise motivation; EC, exercise commitment; EB, exercise behavior.

## Data Availability

The data that support the findings of this study are not openly available but can be made available by the corresponding author upon reasonable request.

## Appendix A. Scale Items

**Variable**		**Item**	**Options**	**Source**
Live Sports Learning	Participation frequency	In general, how often do you participate in online fitness live streaming per week? In general, how often do you participate in sports event live streaming per week? In general, how often do you participate in physical education live streaming per week? In general, how often do you participate in e-sports live streaming per week? In general, how often do you participate in sports e-commerce live streaming per week?	①Less than once ②Once ③Twice ④Three times ⑤More than three times	Dai [47] Guo [48] Zhang [49]
	Participation duration	How much time do you spend on average on online fitness live streaming? How much time do you spend on average on sports event live streaming? How much time do you spend on average on physical education live streaming? How much time do you spend on average on e-sports live streaming? How much time do you spend on average on e-commerce live streaming?	①Within 15 min ②15–30 min ③0.5–1 h ④1–1.5 h ⑤More than 1.5 h	
	Consumption amount	How much money have you spent on online fitness live streaming in the past semester? How much money have you spent on sports event live streaming in the past semester? How much money have you spent on physical education live streaming in the past semester? How much money have you spent on e-sports live streaming in the past semester? How much money have you spent on e-commerce live streaming in the past semester?	Please fill in the Arabic numerals.	
Exercise Motivation	Health motivation	I want to have a strong body. I want to keep my body and mind healthy. I want to live a healthy life.	①No ②A little ③Stronger ④Strong ⑤Very strong	Ryan [33] Chen [51]
	Appearance motivation	I want to control my weight. I want to maintain or improve my body shape. I want to make my appearance more attractive.		
	Social motivation	I want to participate in recreational activities. I want to maintain a happy mood. I want to enjoy a happy life.		
	Ability motivation	I want to acquire new motor skills. I want to improve my current athletic skills. I want to maintain my current level of athletic skills.		
	Fun motivation	I want to meet new people. I want to improve my relationships and friendships with friends. I want to maintain good social relationships		
Exercise Commitment		It was difficult for me to quit physical exercise. If you do not exercise for a few days, you will have a strong desire to participate in physical exercise. It’s hard for me to accept the lack of physical activity. Physical exercise is an indispensable part of my life.	①Strongly disagree ②Disagree ③Neither agree nor disagree ④Agree ⑤Strongly agree	Chen [27]
Exercise Behavior	exercise time	Typically, how long do you participate in physical activity each time?	①Within 15 min ②15–30 min ③0.5–1 h ④1–1.5 h ⑤More than 1.5 h	Liang [52]
	exercise frequency	Normally, how many times per week do you participate in physical activity?	①Less than once ②Once ③Twice ④Three times ⑤More than three times	
	exercise intensity	Normally, what are the changes in your body after completing a physical activity?	①No sensation ②Slightly warm ③Slightly sweaty ④Moderate sweating ⑤Heavy sweating	
